# Apolipoprotein B/A1 Ratio as a Diagnostic Alternative to Triglycerides and HDL-Cholesterol for the Prediction of Metabolic Syndrome among Hypertensives in Kazakhstan

**DOI:** 10.3390/diagnostics10080510

**Published:** 2020-07-23

**Authors:** Alma Nurtazina, Dana Kozhakhmetova, Daulet Dautov, Aizhan Shakhanova, Vijay Kumar Chattu

**Affiliations:** 1 Department of Epidemiology and Biostatistics, Semey Medical University, Semey 071400, Kazakhstan; 2 Department of Propedeutics of Internal Diseases, Semey Medical University, Semey 071400, Kazakhstan; dana_ken@mail.ru (D.K.); aizhanshat@mail.ru (A.S.); 3 Department of Propedeutics of Internal Diseases, Kazakh National Medical University, Almaty 050000, Kazakhstan; daudauda@gmail.com; 4 Department of Medicine, Faculty of Medicine, University of Toronto, Toronto, ON M5S 1A8, Canada

**Keywords:** metabolic syndrome, apolipoprotein B, apolipoprotein A1, triglycerides, high-density lipoprotein cholesterol (HDL-C), smoking, cardiovascular diseases, hypertension, multiple regression, Kazakhstan

## Abstract

Apolipoproteins (Apo) are known atherogenic factors that play important roles in many mechanisms related to coronary heart disease. The ApoB/ApoA1 ratio is a promising diagnostic tool for metabolic syndrome (MS) in different populations, though its use is not established in Kazakhstan. This study aimed to assess the relationship between MS and the ApoB/ApoA1 ratio among hypertensive patients and to evaluate its diagnostic use for identifying MS as an alternative to triglycerides (TG) and high-density lipoprotein cholesterol (HDL-C). A cross-sectional study was conducted in 800 eligible men and women with primary hypertension from April 2015 to December 2016. Data were collected on socio-demographics, lifestyle parameters, family history of cardiovascular disease, and hypertension. Dietary Quality Score (DQS), anthropometric data, and blood pressure were recorded; ApoA1 and ApoB levels were measured in blood samples. We found a significant positive association between MS and the ApoB/ApoA1 ratio by multiple logistic regression, as shown by a linear trend of increase of the odds ratio (OR) for MS across the quartiles of ApoB/ApoA1 (*p* < 0.0001). ROC analysis revealed diagnostic significance of the ApoB/ApoA1 ratio for MS, and comparative ROC analysis demonstrated equal diagnostic value of ApoB/ApoA1 ratio and TG levels (AUC = 0.71 (95% CI 0.69; 0.74) and 0.72 (95% CI 0.69, 0.76), respectively), which was significantly higher than those of HDL, ApoA1, ApoB (AUC = 0.27 (95% CI 0.23; 0.31), AUC = 0.37 (95% CI 0.33; 0.41), AUC = 0.67, (95% CI 0.63; 0.71), respectively). The diagnostic value of the ApoB/ApoA1 ratio in Kazakhs with MS appeared to equal that of TG and was significantly higher than that of HDL-C. Adjusting for gender, smoking, and DQS significantly strengthened the association between MS and the ApoB/ApoA1 ratio in the Kazakh population.

## 1. Introduction

The growing burden of metabolic syndrome (MS), a precursor of cardiovascular diseases (CVD) and diabetes mellitus (DM), currently poses as a serious threat, increasing cardiovascular risk (CVR) and coronary heart disease (CHD) mortality worldwide [1,2,3]. The condition is highly prevalent (range from 11.6% to 49%) and is expected to become more prevalent in the future [4,5,6]. Early identification and treatment of MS components are essential for the prevention and reduction of cardiovascular events and DM. The presence of various ethnic, anthropometric, and genetic differences in the Asian region prompts the adaptation of existing diagnostic MS scales [7,8,9]. Almost all scales and criteria used to determine MS [10,11,12,13] are based on the interpretation of the levels of the lipid profile indicators triglycerides (TG) high-density lipoprotein cholesterol (HDL-C).

However, traditional lipid biomarkers cannot provide sufficient accuracy in the measurement of dyslipidemia. Alongside, transporting molecules like apolipoprotein B (ApoB) are present in all atherogenic types of lipoproteins such as low-density lipoprotein cholesterol (LDL-C), very low density lipoprotein cholesterol (VLDL-C), and intermediate-density lipoproteins (IDL), and their determination enables a more precise estimation of atherogenicity than the conventional lipid profile [14,15]. Apolipoprotein A1 (ApoA1), an antiatherogenic lipoprotein transporting molecule, constitutes a major part of HDL-C [16,17]. ApoA1 is considered a favored alternative to HDL-C in the prediction of cardiovascular diseases, insulin resistance, and DM [18,19,20,21]. Thus, the ApoB/ApoA1 ratio appears to be a more balanced and comprehensive indicator of lipid metabolism and prediction of CVDs, DM, and MS [22,23,24,25].

Several studies have reported the relationship between ApoB/ApoA1 ratio and MS in different ethnic cohorts, including European and South-Asian populations [16,17,26,27]. Nevertheless, the validity of the ApoB/ApoA1 ratio as a potential criterion for MS diagnosis is still unknown for the Central Asian populations. Since there are no data available on the comparative diagnostic significance of ApoB/ApoA1 ratio, TG, and HDL-C for MS in Central Asia, we aimed to investigate the relationship between MS and ApoB/poA1 ratio and to evaluate the possibility of using the ApoB/ApoA1ratio as an alternative to TG and HDL-C for the diagnosis of MS in the Kazakh population.

## 2. Experimental Section

This is an analytical cross-sectional study which included 16 primary-care centers (PHCs) of Semey city in the East Kazakhstan region of the Republic of Kazakhstan. Recruitment and examination were carried out from 6 April 2015 to 31 December 2016. All those who met the inclusion criteria (being a resident of East Kazakhstan and diagnosed with hypertension) were invited for interviews, examination, and laboratory tests. The participants were provided with information about the study objectives and were enrolled after signing the informed consent. The ethical approval of the study was granted by the Ethics Committee (dated 18 February 2015 and Identification No. 0115РК1862) of Semey State Medical University, Ministry of Health of Republic of Kazakhstan. Of the total 800 eligible participants comprising both men and women with a confirmed diagnosis of essential hypertension (HT) [28], aged 25–75 years, only 704 participants who had complete information were included in the study. The exclusion criteria included previous stroke, myocardial infarction, existing diabetes mellitus, hypothyroidism (or taking thyroid hormones), thyrotoxicosis (or taking antithyroid agents), benign and malignant neoplasms, mental illness, pregnant and lactating women, and those who took statins on a regular basis for less than six months before involvement in the study.

### 2.1. Sampling

Two-stage sampling was performed. We randomly selected 16 out of 40 general practices (GP) in Semey city. Then, sample frames were developed for potential participants from every GP based on the lists of hypertensive patients who met the inclusion criteria. In the second stage, we drew out 50 participants from every unit. Randomization was performed by simple random sampling through a computer program for generating random numbers.

### 2.2. Variables

The primary risk factor was Apolipoprotein B/Apolipoprotein A1 ratio; the outcome was metabolic syndrome. The analysis included indicators of the lipid spectrum, i.e., LDL-C, HDL-C, TG, Apo B, and Apo A1. All other factors, such as gender, age, education, income, alcohol use, smoking, physical activity, were considered as potential interfering factors. MS was diagnosed by the International Diabetes Federation Criteria (IDF 2006) and based on blood pressure > 130/85 mmHg, waist circumference > 94 cm in males and > 84 cm in females or body mass index (BMI) > 0 kg/m^2^, hypertriglyceridemia > 1.7 mmol/L, reduced HDL-C < 1.03 mmol/L in males and < 1.29 mmol/L in females, and hyperglycemia–fasting plasma glucose (FPG) > 5.6 mmol/L [9].

### 2.3. Data Collection

All data for each study participant were entered into an individual registration card and coded. Then, a single electronic database was obtained. Confidentiality and privacy of the data were ensured throughout the study, as only the project manager and the data entry operator had access to the electronic database. The database will be stored for up to 5 years, after which it will be destroyed. All participants were asked to fill out a questionnaire, which included socio-demographic indicators (gender, age, education, income), physical activity level, harmful habits (smoking), alcohol consumption, heredity for cardiovascular disease, and hypertension. Data on comorbidity were obtained from the medical records. Dietary assessment was performed by a validated questionnaire, determining the Dietary Quality Score (DQS) [29], translated into Russian. Eating habits were evaluated in three categories: unhealthy (1–4 points), intermediate (4–6 points), and healthy (7–9 points).

### 2.4. Anthropometry Data and Blood Pressure Measurements

Measurement of height and weight was carried out by certified nurses in the pre-doctor room of PHCs according to the recommendations given by the European Society of Hypertension (ESH) and the European Society of Cardiology (ESC) [28]. Height and weight were measured using standardized stadiometer and scale. Measurement of blood pressure (BP) was carried out by the Korotkov method at rest in a sitting position with a membrane sphygmomanometer as per ESC, 2013, page 2168 [28]. We performed two consequent measurements for every participant and used their mean. BMI was classified as by WHO into three categories: normal weigh, <25 kg /m^2^, overweight, 25–29.9 kg /m^2^, and obesity, >30 kg/m^2^.

### 2.5. Laboratory Data

All blood samples were taken in the morning via intravenous venesection after a fasting period of 12 h as a minimum. ApoA1 and ApoB were measured by the immunoturbidimetric method using the analyzer “Cobas 6000”, biochemical module c501, Roche Diagnostics GmbH (Registration certificate No. РК-МТ-712668, Republic of Kazakhstan). The test systems Tina-quant Apolipoprotein A-1 ver.2 and Tina-quant Apolipoprotein B ver.2, manufactured by Roche Diagnostics GmbH, were used. The reference values did not differ from the generally accepted ones. Additionally, the levels of TC, LDL-C, HDL-C, TG, fasting glucose, and glucose tolerance were measured. The glucose tolerance test (GTT) was carried out in a standard manner with 75 g of glucose powder dissolved in 0.5 L of filtered water. Initially the fasting blood sugar levels are measured, and then the glucose solution prepared as mentioned above is given orally after which, the blood is drawn again after two hours to measure the blood sugar levels.

### 2.6. Diagnostic Criteria

The diagnosis of hypertension was made based on the recommendations of the ESC [16] after exclusion of secondary/symptomatic hypertension. All participants had a confirmed diagnosis of essential HT, registered in medical records, regularly took antihypertensive medications, and were followed up by general practitioners.

MS was diagnosed according to IDF 2006 criteria [11], including abdominal obesity or BMI >30 kg/m^2^ plus two of the following four factors: TG > 1.7 mmol/L, HDL-C < 1.03 mmol/L in men and < 1.29 mmol/L in women, BPsyst >130 mmHg or BPdiast > 85 mmHg, plasma glucose > 5.6 mmol/L.

### 2.7. Biases

To minimize the selection bias, we performed a two-level sampling; each level was random and allowed the study group to be representative of the target population. To reduce the measurement error, BP, height, and weight measurements as well as laboratory tests were performed in a single laboratory using standard methods. Interviews were conducted in a standardized way by specially trained team staff to decrease observer bias. MS, HT, and obesity were clearly defined and diagnosed according to established criteria before the study.

### 2.8. Sample Size

Assuming a 95% confidence interval (CI) with 80% power, and ensuring an approximately equal proportion of MS and non-MS subjects in the study group (with a composition of 25% with increased ApoB/ApoA1 ratio among HT patients without MS, and 35% with increased ApoB/ApoA1 ratio among those with MS), we calculated that the total sample size to be 698. Considering the lack of data on the prevalence of elevated levels of ApoB/ApoA1 ratio both in general and among people with MS, as well as anticipating some possible dropouts and self-exit from the study for various reasons, based on the literature review, we recruited a sufficient number of 800 participants in the study.

### 2.9. Quantitative Variables

Indicators of the lipid profile of ApoB/ApoA1, ApoB, ApoA1, TG, LDL-C, HDL-C, TC were employed as quartiles Q1–Q4. MS, gender, alcohol consumption status, gym class attendance, hereditary status for HT and CVD were presented as binary variables. Smoking status was categorized into three groups: not smoking, smoking and quitted. We divided age into five groups (<39, 40–49, 50–59, 60–69, and >70 years old). Both education and income were considered as categorical, ordinal variables.

### 2.10. Statistical Methods

Statistical analysis was performed with Stata Statistical Software: Release 15, College Station, TX: StataCorp LLC. Categorical variables were calculated as proportions (%). Chi-squared test or Fisher’s exact test (for nonparametric variables) were performed between categorical variables and MS for the preliminary association. The odds ratio (OR) with 95% CI was calculated to identify the association between ApoB/ApoA1 ratio and MS by means of logistic regression. We built a multiple logistic regression model by the stepwise forward method. The significance of covariates included in the final logistic regression model was also assessed by a likelihood ratio test (LRT).

ROC analysis was used to assess the diagnostic significance of the ApoB/ApoA1 ratio in MS, as well as the diagnostic value of the final model of multiple logistic regression, taking into account potential confounders and determining cutoff points based on the trade-off between sensitivity and specificity. Also, ROC analysis was used to compare the areas under the ROC curves for the biochemical markers of the lipid spectrum ApoB/ApoA1, ApoB, ApoA1, HDL-C, and TG for the diagnosis of MS before and after adjustment for the potential confounding factors in the ROC regression model.

### 2.11. Participants

Primarily, 800 participants met the inclusion criteria. After additional physical and laboratory examination, some of the participants who were diagnosed with DM and CHD were excluded as per the exclusion criteria. Fifty participants had incomplete laboratory examinations. Also, 10 participants refused to participate in the study. Thus, a total of 704 participants entered the final sample.

## 3. Results

### 3.1. Descriptive Data

Data of 314 men and 390 women were analyzed. Almost half (54.6%) of them met the criteria of MS, of which 62.8% were women, and 37.2% were men (*p* < 0.0001). Of the total 704 subjects, 43.3% were overweight, 36.2% were obese, and only 21% had normal weight.

Table 1 presents data on the distribution of the risk factors for MS by gender, age, education, income, smoking and drinking status, gym class visits, heredity for HT and CVD, and the final DQS score. Gender, smoking, and DQS showed a statistically significant association with MS.

Of the 255 participants with obesity, 74.1% met the criteria of MS, whereas 25.9% did not. Among those with a healthy weight, 144 patients, corresponding to 22.9%, had MS, and 77.1% did not.

Among subjects with MS, 12.2% were smokers, 8.6% were quitters, and 79.2% were non-smokers; 99.2% of females with MS were non-smokers. It was also found that the majority (82%) of the participants had a healthy lifestyle, whereas the remaining (18%) had an intermediate-quality lifestyle.

### 3.2. Components of the Metabolic Syndrome and Other Characteristics of the ApoB/ApoA1 Ratio

Table 2 shows the distribution of the components of MS and other characteristics across quartiles of the ApoB/ApoA1 ratio. The lowest quartile of ApoB/ApoA1 was twice as common in women, and the fourth quartile was more common in men (*p* < 0.001). We found that 60% of the subjects with the highest HDL-C values and 2.3% of those with the lowest one presented the ApoB/ApoA1 ratio in the first quartile (*p* < 0.001). Almost 60% of Q1 and Q4 TG was associated with the Q1 and Q4 of ApoB/ApoA1 ratio, respectively. Nearly half of the participants with a healthy weight had the ApoB/ApoA1 ratio in the lowest quartile, and over one-third (32.6%) of obese cases were associated with ApoB/ApoA1 in Q4. The lowest quartile of ApoB/ApoA1 was predominant for patients with first degree of hypertension (*p* < 0.05).

### 3.3. Association between Metabolic Syndrome (MS) and ApoB/ApoA1 Ratio

Table 3 summarizes the results of the relationship between MS and ApoB/ApoA1 ratio, taking the confounding factors into account. To evaluate the association between MS and ApoB/ApoA1 ratio, we developed a final multiple logistic regression model by means of a stepwise forward approach. There was a linear trend of increase in the crude OR of MS across the quartiles of ApoB/ApoA1 (*p* < 0.0001). Out of all covariates, only gender and DQS were found to be confounding factors for a positive association between MS and ApoB/ApoA1 ratio, making this association stronger across models.

After adjustment, the OR of MS for every quartile of ApoB/ApoA1 became stronger and remained statistically significant.

At fixed values of covariates in the final fitted model of logistic regression, hypertensives in the highest ApoB/ApoA1 ratio quartile had nearly twice the odds of MS compared with those in lowest quartile of ApoB/ApoA1 ratio (OR = 1.99, 95% CI 1.37;3.22, *p* < 0.001). Women had twice the odds of developing MS as men (OR = 2.1, 95% CI 0.31; 0.73, *p* = 0.001). Smokers showed a 32% reduction in the odds of developing MS than non-smokers (OR= 0.68; 95% CI 0.53; 0.89, *p* = 0.005). Subjects with higher DQS demonstrated a 34% decrease in the odds of MS compared with those with lower DQS (OR = 0.66, 95% CI 0.43;1.00, *p* = 0.052).

### 3.4. ROC Analysis, Predictive Value of a Fitted Model of Logistic Regression Including Covariates

**Figure 1:**
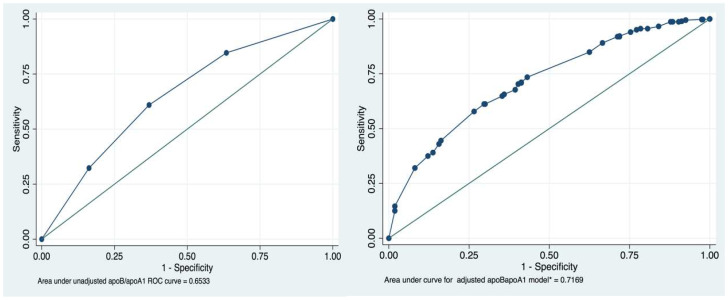
Receiver operating characteristic (ROC) curves and area under the curve (AUC) for the ApoB/ApoA1 ratio in unadjusted and adjusted regression logistic models for the association between MS and ApoB/ApoA1 ratio.

**Figure 2:**
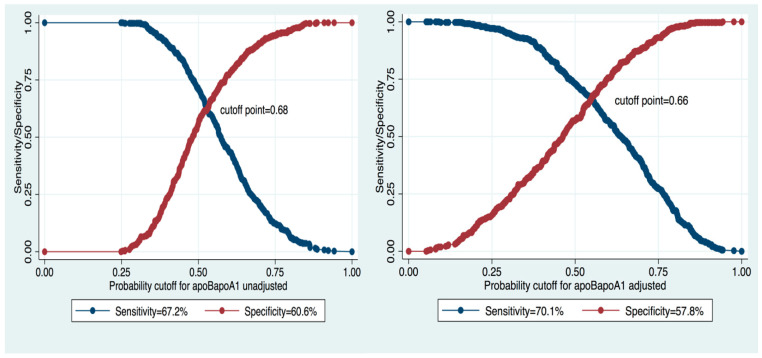
Cutoff point, sensitivity, and specificity of Apo/ApoA1 ratio in unadjusted and adjusted models.

To assess the predictive value of the ApoB/ApoA1 ratio for MS diagnosis, we performed a ROC analysis of the fitted logistic regression model (Figure 1). We evaluated the model using the ROC curve before and after adjustment for potential confounding factors. After adjustment for gender, DQS, and smoking, the area under the curve (AUC) increased from 0.67 to 0.72. The cut-off point for the ApoB/ApoA1 ratio decreased from 0.68 (sensitivity 67.2%, specificity 60.6%) to 0.66 (sensitivity 70.1%, specificity 57.8%) (Figure 2).

**Figure 3:**
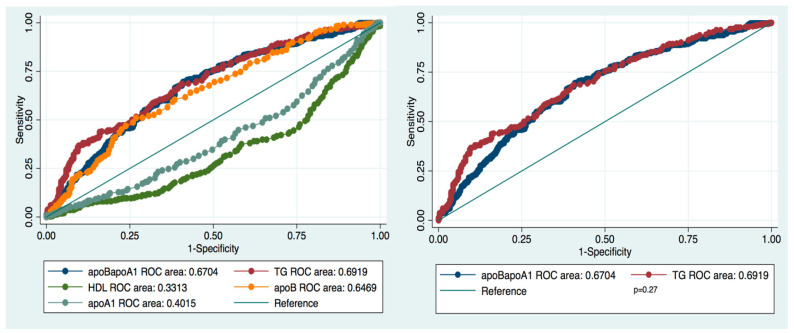
Comparison of ROC curves and AUCs of ApoB/ApoA1, TG, HDL-C, ApoB, ApoA1 for the diagnosis of MS.

We compared the values of TG and HDL-C with those of the ApoB/ApoA1 ratio and of its components ApoB and ApoA1 as criteria for MS diagnosis (Figure 3). The ApoB/ApoA1 ratio and TG demonstrated to be the best predictive parameters of MS, with very similar ROCs (AUC_ApoB/ApoA1_ = 0.67, 95% CI 0.63; 0.71; AUC_TG_ = 0.69, 95% CI 0.65; 0.73, *p*= 0.27, Sidak correction *p* = 0.72).

The diagnostic value of HDL-C appeared to be unreliable because of the small AUC (0.27 vs 0.72 and 0.71 for HDL-C, TG, and ApoB/ApoA1, respectively). After adjustment, 71% of the time, patients with MS had a higher ApoB/ApoA1 ratio than those without MS. Adjusting for gender, DQS, and smoking enhanced the AUC for TG, ApoB/ApoA1 ratio, and ApoB and decreased the AUC for HDL-C and apoA1 (Table 4).

Table 5 presents data on estimated cutoff values and the validity of lipid biomarkers for the diagnosis of MS before and after adjustment. TG and the ApoB/ApoA1 ratio showed the highest sensitivity and specificity of their cutoff levels before and after adjustment for gender and age, while HDL-C sensitivity and specificity were the lowest.

## 4. Discussion

### 4.1. ApoB/ApoA1 Ratio vs. TG and HDL-C

In this study, we found a significant association between MS and ApoB/ApoA1 ratio in the Kazakh population. We also observed a linear trend of the OR of MS across quartiles of the ApoB/ApoA1 ratio (*p* trend = 0.0001). The highest ORs of MS were estimated in Q4 of the ApoB/ApoA1 ratio both before and after adjustment for potential confounding factors, nearly four- and eight-fold higher versus those in Q1, respectively. These findings are in agreement with previous studies [24,25,26,30,31].

ApoB and ApoA1 are transporting components of atherogenic (LDL-C, VLDC-C, and IDL) and antiatherogenic (HDL-C) lipid profiles, respectively. Abundant evidence has proven a higher predictive ability of the ApoB/ApoA1 ratio for CVDs [24,30], obesity, insulin resistance [20,21,27], and diabetes [22,31] over conventional biomarkers. The ApoB/ApoA1 ratio is linked to early atherosclerosis as well [23]. In turn, MS is associated with 1.5–3-times higher CVR and CHD mortality and about a 5-times higher risk for DM.

Currently, there are very few studies in the literature focused on the value of the ApoB/ApoA1 ratio for the diagnosis of MS in various ethnic populations, including Chinese, Greek, Korean, Taiwan Chinese, Tunisian, and Bulgarian ethnicities. The predictive value of the ApoB/ApoA1 ratio was compared mostly with those of LDL-C, HDL-C, ApoB, and apoA1 [26,27,32,33,34]. None of the previous studies evaluated the ApoB/ApoA1 ratio versus the level of TG as a standard laboratory criterion for MS diagnosis. In our research, based on ROC analysis, we estimated almost equal MS diagnostic performances for the ApoB/ApoA1 ratio and TG (AUC = 0.72 and AUC = 0.71, respectively). In contrast, HDL-C, another standard laboratory parameter for MS diagnosis, demonstrated poor diagnostic accuracy (AUC = 0.27).

Hypertriglyceridemia and decreased HDL-C level, as laboratory criteria for MS diagnosis, characterize atherogenicity and are associated with increased risk of CVD. Additionally, hypertriglyceridemia could be the result of insulin resistance. Nevertheless, recent data did not confirm the role of isolated hypertriglyceridemia in the prediction of CVR. As authors mentioned, this criterion could be valid only when combined with the levels of LDL-C and HDL-C [35,36,37]. Also, some authors discuss technical inconveniences related to the requirement of a 12 h fast before taking blood sample for TG and HDL-C analysis [30]. In contrast, the measurement of ApoB and ApoA1 does not depend on fasting. Thus, the ApoB/ApoA1 ratio appears to be more valid and suitable in clinical settings than TG and HDL-C levels for the identification of subjects with MS.

In the present study, we estimated that 0.66 (sensitivity 70.1%, specificity 57.8%) and above is an adjusted optimal cutoff point for the ApoB/ApoA1 ratio for discriminating subjects with and without MS after controlling for potential confounding factors in the Kazakh population. The value we determined is similar to that for the Korean population (cutoff = 0.65 [32]) but lower than those for the Chinese (cutoff = 0.82) [27,30] and Greek populations (cutoff = 0.72) [38]. These differences can be probably explained by a lower prevalence of MS and/or a lower mean ApoB in Kazakhs compared to Chinese and Greeks. Further research on larger samples is warranted to confirm our study results.

### 4.2. Limitations

The study has its own limitations which should be taken into account when interpreting the results. Firstly, the study population included only hypertensives, who do not adequately represent the general population. None of the participants were newly diagnosed patients with hypertension but had been monitored regularly by general practitioners for one to two decades, were on medical therapy and were following a healthy lifestyle. Thus, the prevalence of MS and dietary habits in our research cannot be fully generalized. The finding of a “preventive” effect of smoking could also be a result of methodological constraints in our study. Lastly, the optimal cutoff point of the ApoB/ApoA1 ratio was not estimated separately for men and women.

## 5. Conclusions

The diagnostic value of the ApoB/ApoA1 ratio in Kazakhs for MS equals that of TG and is significantly higher than that of HDL-C. Adjusting for gender, smoking, and DQS significantly strengthened the association between MS and the ApoB/ApoA1 ratio in the Kazakh population. Further large-scale studies are warranted to confirm the results of our study, determine the accuracy of ApoB/ApoA1 ratio for the diagnosis of MS, and clarify the role of gender, smoking, and dietary habits.

## Figures and Tables

**Table 1:** Basic demographic and clinical characteristics of the study population (N = 704).

Characteristics	Frequency Distribution		MS, %		*p*-Value
	Absolute Number	%	Yes	No	
**Gender:**					0.0001
male	314	44.6	37.2	53.4	
female	390	55.4	62.8	46.6	
age (years):					0.02, 0.01 *
<39	71	10.1	8.1	12.5	
40–49	216	30.7	31.5	29.7	
50–59	232	33.0	35.2	30.3	
60–69	154	21.9	22.7	20.9	
>70	31	4.4	2.6	6.6	
Education:					0.43
Secondary school	92	13.1	14.6	11.3	
College	393	55.8	55.0	56.9	
University	219	31.1	30.5	31.9	
Income, KZT/capita/month:					0.52
<50,000	329	46.7	48.4	44.7	
50,000–100,000	351	49.9	47.9	52.2	
>100,000	24	3.4	3.7	3.1	
Smoking:					0.0001, 0.0001 *
No	511	72.6	79.2	64.7	
Quit	61	8.7	8.6	8.8	
Yes	132	18.8	12.2	26.6	
Alcohol consumption:					0.56
No	411	58.4	59.4	57.2	
Yes	293	41.6	40.6	42.8	
Gym class					0.48
No	631	89.6	90.4	88.8	
Yes	73	10.4	9.6	11.2	
Hereditary for hypertension					0.03
No	292	41.7	38.1	46.1	
Yes	408	58.3	61.9	53.1	
Hereditary for CHD					0.11
No	546	76.6	75.3	80.3	
Yes	158	22.4	24.7	19.3	
GTT					0.0001
normal	420	77.8	68.2	90.2	
abnormal	120	22.2	31.8	9.8	
DQS					0.01
Score ≤6	126	17.9	14.6	21.9	
Score > 6	578	82.10	85.4	78.1	

* *p* for trend of odds; CHD, coronary heart disease; GTT, glucose tolerance test; DQS, Diet Questionnaire Score.

**Table 2:** Characteristics across quartiles of ApoB/apoA1 ratio.

Variables	Quartiles of ApoB/ApoA1				100%	*p*-Value
	Q1 (<0.56)	Q2 (0.57–0.70)	Q3 (0.71–0.84)	Q4 (≥0.85)		
Gender						0.001
Male	16.2	23.3	24.2	36.3	100%	
Female	32.1	26.4	25.6	15.9	100%	
Smoking						0.001
no	28.0	27.0	24.7	20.4	100%	
quitted	13.1	20.0	24.6	42.6	100%	
yes	18.9	19.7	26.5	34.9	100%	
Degree of hypertension						0.05
1	30.6	27.2	17.5	24.8	100%	
2	23.7	23.4	28.7	24.2	100%	
3	19.7	26.2	26.2	27.9	100%	
HDL-C, quartiles (mmol/L)						0.001
Q1 min/1.14	2.3	9.8	31.0	56.9	100%	
Q2 1.15/1.37	10.7	29.2	32.0	28.1	100%	
Q3 1.38/1.67	27.3	34.3	26.7	11.6	100%	
Q4 1.68/max	58.9	26.7	10.6	3.9	100%	
TG, quartiles (mmol/L)						0.001
Q1 min/0.85	56.7	27.8	12.7	2.9		
Q2 0.85/1.14	24.2	34.1	28.6	13.2		
Q3 1.14/1.69	15.3	26.7	31.8	26.1		
Q4 ≥1.7	4.1	11.0	26.6	58.4		
BMI categories (kg/m^2^)						0.001
<24.9	42.4	22.9	22.2	12.5	100%	
25.0–29.9	23.0	27.5	24.9	24.6	100%	
≥30	17.7	23.1	26.7	32.6	100%	
DQS						0.5
≤6	24.6	26.2	20.6	28.6		
>6	25.1	24.7	26.0	24.2		

HDL-C, high-density lipoprotein cholesterol, TG, triglycerides, BMI, body mass index.

**Table 3:** Crude and adjusted odds ratios (ORs) of metabolic syndrome (MS) for 25, 50, 75% quartiles of the ApoB/ApoA1 ratio.

Number of Observations	Quartiles of ApoB/ApoA1 Ratio				Adjusted for	Model	*p*-Value of Trend
	Q1 (<0.57)	Q2 (0.57/0.7)	Q3 (0.71/0.85)	Q4 (>0.85)			
704	baseline	2.12 ^†^ (1.38; 3.2)	3.31 ^††^ (2.13; 5.12)	4.73 ^††^ (3.01; 7.42)	Crude	1	0.0001
704	baseline	2.57 ^††^ (1.64; 4.03)	4.23 ^††^ (2.67; 6.72)	7.94 ^††^ (4.81; 13.11)	Gender	2	
704	baseline	2.60 ^††^ (1.65; 4.08)	4.21 ^††^ (2.64; 6.69)	8.12 ^††^ (4.90; 13.46)	Gender + DQS	3	
704	baseline	2.5 3 ^††^ (1.61; 3.98)	4.29 ^††^ (2.69; 6.85)	8.31 ^††^ (4.99; 13.84)	Gender * + DQS ^ψ^ + Smoking ^ϒ^	4	

^†^ Wald *p* < 0.001; ^††^ Wald *p* < 0.0001; * likelihood ratio test (LRT); gender *p* = 0.0005; ^ψ^ LRT DQS *p* = 0.05; ^ϒ^ LRTsmoking *p* = 0.001.

**Table 4:** Unadjusted and adjusted ROC areas for TG, ApoB/ApoA1, ApoB, and HDL-C for MS diagnosis.

Variable	Crude AUC (95% CI) Continues	*p*-Value *^	Adjusted *** AUC (95% CI) Continues Gender, DQS, Smoking
TG (reference)	0.69 (0.65; 0.73)	-	0.72 (0.69; 0.76)
ApoB/ApoA1	0.67 (0.63; 0.71)	0.27 * 0.72 ^	0.71 (0.69; 0.74)
ApoB	0.65 (0.61; 0.69)	0.02 * 0.08 ^	0.67 (0.63; 0.71)
ApoA1	0.40 (0.36; 0.44)	0.0001 *^	0.37 (0.33; 0.41)
HDL-C	0.33 (0.29; 0.37)	0.0001 *^	0.27 (0.23; 0.31)

* *p*-value standard. ^ *p*-value by Sidak correction. *** After adjustment for gender, DQS, and smoking.

**Table 5:** Sensitivity and specificity of ApoB/ApoA1 ratio, TG, HDL-C, ApoB, and ApoA1 in the diagnosis of MS.

	Cut off Point	Crude		Cut off Point	Adjusted *	
		Sensitivity (%)	Specificity (%)		Sensitivity (%)	Specificity (%)
TG	1.05	69.27	55.31	1.01	72.92	52.19
ApoB/ApoA1	0.68	67.19	60.62	0.66	70.05	57.81
ApoB	0.98	65.10	55.0	0.96	67.71	51.25
ApoA1	1.46	40.89	44.06	1.49	37.50	48.12
HDL-C	1.45	33.85	43.44	1.51	27.60	49.07

* Adjusted for age and sex.

## References

[1] Hess P.L., Al-Khalidi H.R., Friedman D.J., Mulder H., Kucharska-Newton A., Rosamond W.R., Lopes R.D., Gersh B.J., Mark D.B., Curtis L.H. (2017). The metabolic syndrome and risk of sudden cardiac death: The atherosclerosis risk in communities study. J. Am. Heart Assoc..

[2] Motillo S., Filion K., Genes J., Joseph L., Pilote L., Poirier P., Rinfret S., Schiffrin E., Eisenberg M. (2010). The metabolic syndrome and cardiovascular risk. A systematic review and meta-analysis. J. Am. Coll. Cardiol..

[3] Wilson P.W., D’Agostino R.B., Parise H., Sullivan L., Meigs J.B. (2005). Metabolic syndrome as a precursor of cardiovascular disease and type 2 diabetes mellitus. Circulation.

[4] van Vliet-Ostaptchouk J.V., Nuotio M.L., Slagter S.N., Doiron D., Fischer K., Foco L., Gaye A., Gögele M., Heier M., Hiekkalinna T. (2014). The prevalence of metabolic syndrome and metabolically healthy obesity in Europe: A collaborative analysis of ten large cohort studies. BMC Endocr. Disord..

[5] Ford E.S., Giles W.H., Dietz W.H. (2002). Prevalence of the metabolic syndrome among US adults—Findings from the third national health and nutrition examination survey. JAMA.

[6] Ranasinghe P., Mathangasinghe Y., Jayawardena R., Hills A.P., Misra A. (2017). Prevalence and trends of metabolic syndrome among adults in the asia-pacific region: A systematic review. BMC Public Health.

[7] Zhao Y., Yan H., Yang R., Li Q., Dang S., Wang Y. (2014). Prevalence and determinants of metabolic syndrome among adults in a rural area of Northwest China. PLoS ONE.

[8] Lee W.Y., Park J.S., Noh S.Y., Rhee E.J., Kim S.W., Zimmet P.Z. (2004). Prevalence of the metabolic syndrome among 40,698 Krean metropolitan subjects. Diabetes Res. Clin. Pract..

[9] Yamagishi K., Iso H. (2017). The criteria for metabolic syndrome and the national health screening and education system in Japan. Epidemiol. Health.

[10] Grundy S.M., Cleeman J.I., Merz C.N.B., Brewer H.B., Clark L.T., Hunninghake D.B., Pasternak R.C., Smith S.C., Stone N.J., Coordinating Committee of the National Cholesterol Education Program (2004). Implications of recent clinical trials for the National Cholesterol Education Program Adult Treatment Panel III guidelines. Circulation.

[11] Alberti K.G., Zimmet P., Shaw J. (2006). Metabolic syndrome—A new world-wide definition. A Consensus Statement from the International Diabetes Federation. Diabet. Med..

[12] Balkau B., Charles M.A. (1999). Comment on the provisional report from the WHO consultation. European Group for the Study of Insulin Resistance (EGIR). Diabet. Med..

[13] World Health Organization (1999). Definition, Diagnosis and Classification of Diabetes Mellitus and Its Complications: Report of a WHO Consultation. Part 1, Diagnosis and Classification of Diabetes Mellitus.

[14] Sniderman A.D., Pedersen T., Kjekshus J. (1997). Putting lowdensity lipoproteins at center stage in atherogenesis. Am. J. Cardiol..

[15] Kaneva A.M., Potolitsyna N.N., Bojko E.R., Odland J.Ø. (2015). The apolipoprotein B/apolipoprotein A-I ratio as a potential marker of plasma atherogenicity. Dis. Markers.

[16] Ramasamy I. (2018). Update on the laboratory investigation of dyslipidemias. Clin. Chim. Acta.

[17] Oloffson S.-O., Boren J. (2005). Apolipoprotein B: A clinically important apolipoprotein which assembles atherogenic lipoproteins and promotes the development of atherosclerosis. J. Intern. Med..

[18] Walldius G., Jungner I., Holme I., Aastveit A.H., Kolar W., Steiner E. (2001). High apolipoprotein B, low apolipoprotein A-I, and improvement in the prediction of fatal myocardial infarction (AMORIS study): A prospective study. Lancet.

[19] Makaridze Z., Giorgadze E., Asatiani K. (2014). Association of the apolipoprotein B/apolipoprotein A-I ratio, metabolic syndrome components, total cholesterol, and low-density lipoprotein cholesterol with insulin resistance in the population of Georgia. Int. J. Endocrinol..

[20] Ying X., Qian Y., Jiang Y., Jiang Z., Song Z., Zhao C. (2012). Association of he apolipoprotein B/apolipoprotein A-I ratio and low-density lipoprotein cholesterol with insulin resistance in a Chinese population with abdominal obesity. Acta Diabetol..

[21] Jian Z.H., Lung C.C., Ko P.C., Sun Y.H., Huang J.Y., Ho C.C., Ho C.Y., Chiang Y.C., Chen C.J., Liaw Y.P. (2013). The association between the apolipoprotein A1/high density lipoprotein -cholesterol and diabetes in Taiwan—A cross-sectional study. BMC Endocr. Disord..

[22] Panayiotou A., Griffin M., Georgiou N., Bond D., Tyllis T., Tziakouri-Shiakalli C., Fessas C., Nicolaides A. (2008). ApoB/ApoA1 ratio and subclinical atherosclerosis. Int. Angiol..

[23] Raitakari O.T., Mäkinen V.P., McQueen M.J., Niemi J., Juonala M., Jauhiainen M., Salomaa V., Hannuksela M.L., Savolainen M.J., Kesäniemi Y.A. (2013). Computationally estimated apolipoproteins B and A1 in predicting cardiovascular risk. Atherosclerosis.

[24] Sierra-Johnson J., Somers V.K., Kuniyoshi F.H.S., Garza C.A., Isley W.L., Gami A.S., Lopez-Jimenez F. (2006). Comparison of apolipoprotein-B/apolipoprotein-AI in subjects with versus without the metabolic syndrome. Am. J. Cardiol..

[25] Sniderman A.D., Wolfson C., Teng B., Franklin F.A., Bachorik O.S., Kwiterovich P.O. (1982). Association of hyperapobetalipoproteinemia with endogenous hypertriglyceridemia and atherosclerosis. Ann. Intern. Med..

[26] Belfki H., Ali S.B., Bougatef S., Ahmed D.B., Haddad N., Jmal A., Abdennebi M., Romdhane H.B. (2011). The apolipoprotein B/apolipoprotein A 1 ratio in relation to metabolic syndrome and its components in a sample of the Tunisian population. Exp. Mol. Pathol..

[27] Naydenova G.A., Marinov M.S., Atanasov M.A., Kostadinova P.S., Kostadinov S.D., Tsveova R.S. (2016). Study of the association between interrelation apoB/apoA1,TC/HDL-C, LDL-C and triglyciride/HDL-C and metabolic syndrome among Bulgarian population. Med Sci..

[28] Mancia G., Fagard R., Narkiewicz K., Redon J., Zanchetti A., Böhm M., Christiaens T., Cifkova R., De Backer G., Dominiczak A. (2013). 2013 ESH/ESC guidelines for the management of arterial hypertension: The task force for the management of arterial hypertension of the European Society of Hypertension (ESH) and of the European Society of Cardiology (ESC). Blood Press..

[29] Toft U., Kristoffersen L.H., Lau C., Borch-Johnsen K., Jørgensen T. (2007). The dietary quality score: Validation and association with cardiovascular risk factors: The Inter99 study. Eur. J. Clin. Nutr..

[30] Renee Ruhaak L., van der Laarse A., Cobbaert C.M. (2019). Apolipoprotein profiling as a personalized approach to the diagnosis and treatment of dyslipidaemia. Ann. Clin. Biochem..

[31] Thompson A., Danesh J. (2006). Associations between apolipoprotein B, apolipoprotein A1, the apolipoprotein B/A1 and coronary heart disease: A literature-based meta-analysis of prospective studies. J. Intern. Med..

[32] Pitsavos C., Panagiotakos D.B., Skoumas J., Papadimitriou L., Stefanadis C. (2008). Risk stratification of apolipoprotein B, apolipoprotein A1, and apolipoprotein B/AI ratio on the prevalence of the metabolic syndrome: The ATTICA study. Angiology.

[33] Jung C.H., Hwang J.Y., Yu J.H., Shin M.S., Bae S.J., Park J.Y., Kim H.K., Lee W.J. (2012). The value of apolipoprotein B/A1 ratio in the diagnosis of metabolic syndrome in a Korean population. Clin. Endocrinol..

[34] Chou Y.C., Kuan J.C., Bai C.H., Yang T., Chou W.Y., Hsieh P.C., You S.L., Hwang L.C., Chen C.H., Wei C.Y. (2014). Predictive value of serum apolipoprotein B/apolipoprotein A-I ratio in metabolic syndrome risk: A Chinese cohort study. Endocrine.

[35] Andersson C., Lyass A., Vasan R.S., Massaro J.M., D’Agostino R.B., Robins S.J. (2014). Long-term risk of cardiovascular events across a spectrum of adverse major plasma lipid combinations in the framingham heart study. Am. Heart J..

[36] Bansal S., Buring J.E., Rifai N., Mora S., Sacks F.M., Ridker P.M. (2007). Fasting compared with nonfasting triglycerides and risk of cardiovascular events in women. JAMA.

[37] Miller M., Cannon C., Murphy S., Qin J., Ray K., Braunwald E. (2008). Impact of triglyceride levels beyond low-density lipoprotein cholesterol after acute coronary syndrome in the PROVE IT-TIMI 22 trial. J. Am. Coll. Cardiol..

[38] Fonseca L., Paredes S., Ramos H., Oliveira J.C., Palma I. (2020). Apolipoprotein B and non-high-density lipoprotein cholesterol reveal a high atherogenicity in individuals with type 2 diabetes and controlled low-density lipoprotein-cholesterol. Lipids Health Dis..

